# A Computer-Vision-Guided Robot Arm for Automatically Placing Grids in Pioloform Film Preparation

**DOI:** 10.3390/mps2010009

**Published:** 2019-01-17

**Authors:** Markus Peurla, Pekka E. Hänninen, Eeva-Liisa Eskelinen

**Affiliations:** Institute of Biomedicine, University of Turku, Kiinamyllynkatu 10, FI-20520 Turku, Finland; pekka.e.hanninen@utu.fi (P.E.H.); eeva-liisa.eskelinen@utu.fi (E.L.E.)

**Keywords:** transmission electron microscopy (TEM), grid, film, formvar, pioloform, robot, computer vision

## Abstract

Preparing pioloform/formvar support films on transmission electron microscopy (TEM) grids is a routine laboratory procedure in practically all electron microscopy units. In current practice, these grids are manually placed on the support film one by one using special tweezers, a process requiring a steady hand. The work is often ergonomically awkward to continue for a longer period of time. In this article, we describe a low-cost, computer vision-guided robot arm that automatically places the grids on the film. The success rate of the prototype robot is 90%, which is comparable to an experienced laboratory technician.

## 1. Introduction

Ultrathin sections prepared for transmission electron microscopy (TEM) imaging are picked up on metal grids, which are usually covered by a thin polymer film of some tens of nanometers thickness to give extra support [1]. The bare grids can be obtained commercially, but the film is often prepared in-house because the film casting is an inexpensive and relatively straightforward procedure, and the film properties can be controlled upon need. The method is well established. A clean microscope glass slide is immersed in the film casting solution and lifted up with a steady speed. In this way, a thin polymer film is formed on the complete slide surface. The film is removed from the edges and from one side of the glass slide with a tissue or by cutting with a razor blade. By slowly immersing the slide in ultrapure water at an angle of 45°–90°, the thin film detaches from the slide and floats on the water surface. After this, the grids are carefully placed on the film one by one using tweezers or a dedicated vacuum pick-up system. Placing the grids on the film typically takes several minutes, with the work requiring a steady hand and good concentration in an ergonomically awkward position. Finally, the film containing the grids is lifted out of the water using, for example, a piece of parafilm, paper, or a glass coverslip. The grids can be dried on a hot plate and are generally coated with a thin layer of carbon in order to make the film conductive.

For decades, various robots have been used to replace humans in procedures involving repetitive tasks, especially in industries but also in research laboratories. Recent progress in small, easy-to-deploy, and cheap, yet relatively powerful mini computers, such as the Raspberry Pi, together with extensive open-source software libraries, has enabled straightforward construction of inexpensive devices with complex functionality. These robots are often vision-guided, getting their control information from the surrounding physical world using a camera and pattern or feature recognition algorithms to locate objects [2]. These types of robots can perform complex pick-and-place type tasks, for example, on randomly scattered or oriented objects.

In this article, we describe the concept and prototype construction of a computer-vision-guided robot arm that can automatically place grids on a pioloform film, setting the laboratory technician free to do other preparatory steps. The grids may be given to the robot as a heap on filter paper, and the robot spreads them and places single grids on the film in a desired pattern. The robot is inexpensive as it can be constructed using readily available parts and open-source software libraries. 

## 2. Construction of the Robot

### 2.1. General Design

**Figure 1:**
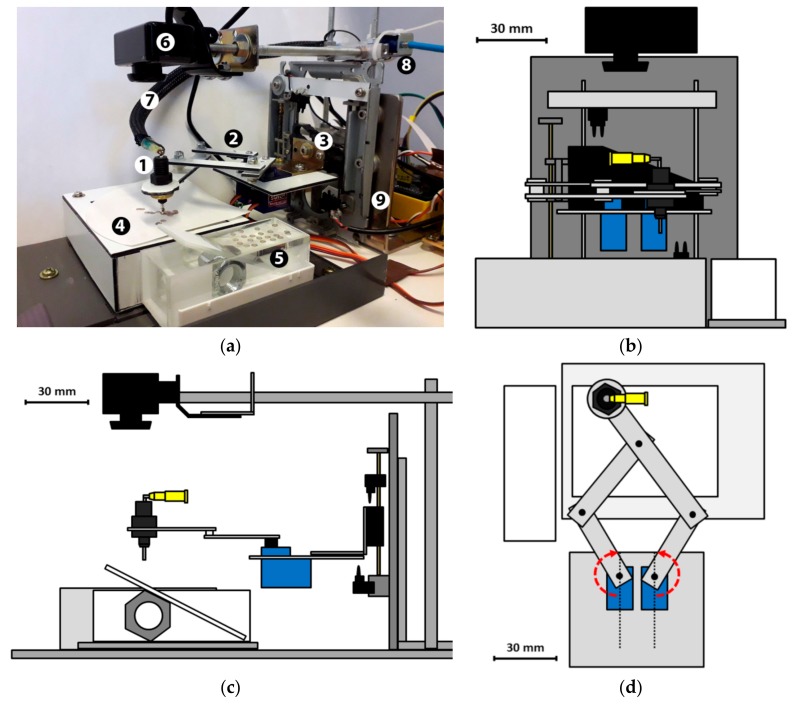

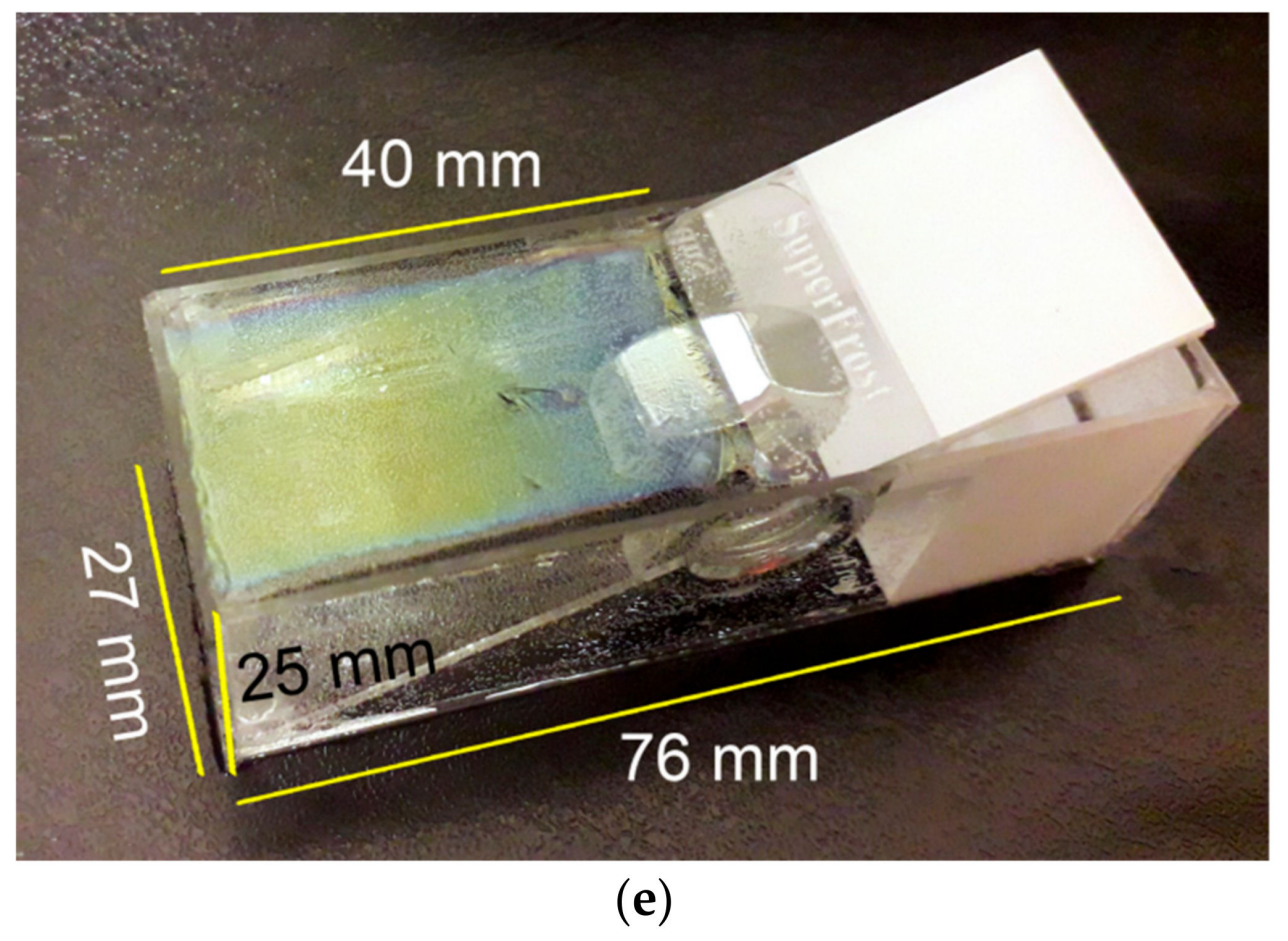
(**a**) Photograph of the grid-placer robot prototype. The main parts are (1) pick-up nozzle, (2) lateral (*x*-*y*) moving arm mechanism, (3) arm height (*z*) moving mechanism, (4) grid pick-up table, (5) water trough for holding the film, (6) web camera with adjustable mount for computer vision, (7) suction line, (8) solenoid valve, and (9) L-bracket attaching the robot to base plate. The suction pump is located outside the image. Schematic (**b**) front, (**c**) side, and (**d**) top views of the prototype. Scale of the schematic drawings is indicated by the scale bars. (**e**) Photograph of the water trough for floating of the film during grid placement. A pioloform film of size 25 mm × 40 mm (seen with the yellow-blue interference color) is confined by the trough and exactly covering the free water surface area, thus being immobilized.

The robot prototype is shown in Figure 1a, and its actions are shown in Supplementary Materials, Video S1. The main components of the system are (1) a suction-operated pick-up nozzle, (2) a lateral (*x*-*y*) moving arm mechanism, (3) an arm height (*z*) moving mechanism, (4) a grid pick-up table, (5) a water trough for holding the film, (6) a web camera with adjustable mount for computer vision, and (7) suction-producing pump and valves. The robot functions are controlled by a Raspberry Pi 3 Model B+ (Premier Farnell Ltd., Leeds, UK). The essential parts with their approximate prices in Euro are listed in Table 1. Because of the camera feedback-based control algorithm, the exact design of the chassis and support structures is not critical and may be modified based on available braces and other parts. Schematic images of our design are shown in Figure 1b–d.

In the manual grid-making procedure, the film is floated off from the microscope slide to the water surface in, for example, a petri dish or a beaker, where it can slowly drift during grid placement. The lab technician can place grids on a slowly moving film without problems; however, in our robot operation logic, the film floating on the water surface needs to be stationary because grids are placed in a predetermined lattice of arm coordinates without any feedback. This is accomplished by building a trough that has the water surface area matching the film size (25 mm wide to 40 mm length) (Figure 1e). The trough is built from microscope slides by gluing them together with Loctite Glass Super Glue. 

### 2.2. Mechanisms

The *x*-*y* arm design is based on a five-bar mechanism [3] that is modified by extending one of the arms. The arm movement is actuated by two TowerPro SG92R servo motors that each has a maximum movement of 180° (see red arrows in Figure 1d). The two arms made of 2-mm-thick aluminum composite sheet (Dilite) are mounted on the servos such that they rotate between the fully forward and fully backward pointing positions without colliding with each other (Figure 1d). This maximizes the robot arm reach area. The geometrical transform for the arm control used by the software is presented in Appendix A. The *x*-*y* servo motors can repeat a given nozzle position with approximately 1 mm accuracy.

The arm height (*z*) movement mechanism is built using a head-moving mechanism of an old DVD drive. It suits this purpose very well because it has a readily sturdy frame and a stepper-motor-operated linear movement mechanism. The robot arm assembly is directly attached to the carriage using an L-shaped bracket. The whole robot stands on a base plate attached by another L-bracket (Figure 1a).

The *z*-movement range is controlled by adjustable limit switches at the top and bottom extremes. The upper limit is adjusted to a position where it stops the nozzle at about 15 mm above the grid table level. The grid pick-up and placement are done at the same *z*-height, which is controlled by the lower limit switch. The trough and grid table are mounted so that their surfaces are at the same level. Any small mismatch (within 1–2 mm) can be corrected by adjusting the water level in the trough. During the grid placement, the imprecision on the limit-switching movement is compensated by the water and film resilience under the grid when the nozzle presses the grid on the film. Pressing the grid on the film, instead of dropping the grid from a height, improves the adhesion between the two. It also ensures that the grid is detached from the nozzle. Occasionally, a grid may remain stuck on the nozzle when grids picked up from a heap are dropped to make them spread apart. A quick up-and-down shake function is made to drop those grids from the nozzle. The correct height of the lower limit switch and the water level in the trough are determined after the robot is put together. The user can control the force by which the nozzle presses the grids on the film by adjusting the water level. It should be strong enough to ensure proper attachment of the grids but not so strong as to damage the film. In our experience, the pioloform film is relatively strong and does not easily rupture when a grid is pressed on it.

### 2.3. Pick-up Nozzle

The robot uses a vacuum pick-up system to lift the grids. A modified Elite 802 diaphragm aquarium air pump (Hagen Inc., Montreal, QC, Canada) is used to generate the suction. The function of the pump is reversed by attaching a hose connector to the air inlet of the pump. Suction is controlled by a 6 V magnetic valve, and it is necessary to have a check valve in the suction line to prevent air back-flow that disrupts the attachment of grids on the nozzle.

A nozzle diameter of 0.9 mm was found optimal for the grid pick-up. We used a modified syringe needle with a Luer connector (Terumo 20G; Terumo, Tokyo, Japan) for the nozzle by carefully cutting or polishing it to 35 mm length and bending it to an angle of approximately 90°, making sure not to distort or flatten the pipe by our modification. 

**Figure 2:**
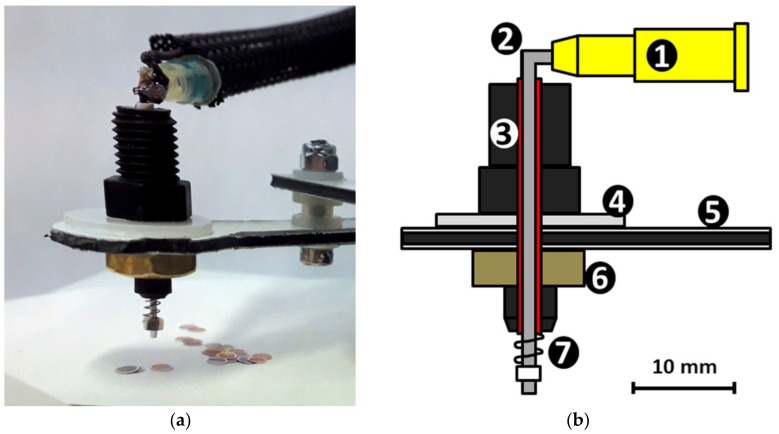
(**a**) Design of the pick-up nozzle. (**b**) Schematic view of the nozzle drawn to scale indicated by the scale bar. The main parts are (1) Luer connector of the needle, (2) needle bent to 90° angle, (3) microfluidic connector, (4) spacer, (5) robot arm, (6) nut, and (7) spring. The wire insulation sheath surrounding the needle is indicated in red.

The construction of the pick-up nozzle assembly is shown in Figure 2. The needle is mounted on the arm through a plastic microfluidic connector (Elvesys, Paris, France) (number 3 in Figure 2b) and a wire insulation sheath (red in Figure 2b) to make fitting exact but allowing a small *z*-movement. It is important that the needle can move freely up and down through the wire sheath in the microfluidic connector as it needs to slide upward when touching the grid table surface to prevent pressing too hard on the grids. A small spring is inserted on the nozzle to bring it back down after sliding up. To make the touch on the grids even more gentle, the grid table top has an edge-frame that is 1 mm higher than the surface of the robot working area so that the filter paper is supported only from the edges. The elastic movement of the paper ensures a more gentle contact between the nozzle and the paper, compared to pressing against a solid surface, when picking up the grids.

The nozzle piece is connected to the solenoid valve by a pneumatic tube measuring 4 mm in diameter. The rest of the suction line from solenoid valve to check valve and to the pump is constructed using the same 4 mm pneumatic tube and 10 mm silicon hose. The tubing is not critical, and any size fitting the existing connectors can be used. Because static electricity on the filter paper sometimes interferes with grid liftoff, the nozzle is grounded by soldering a grounding wire to it.

### 2.4. Electronics

**Figure 3:**
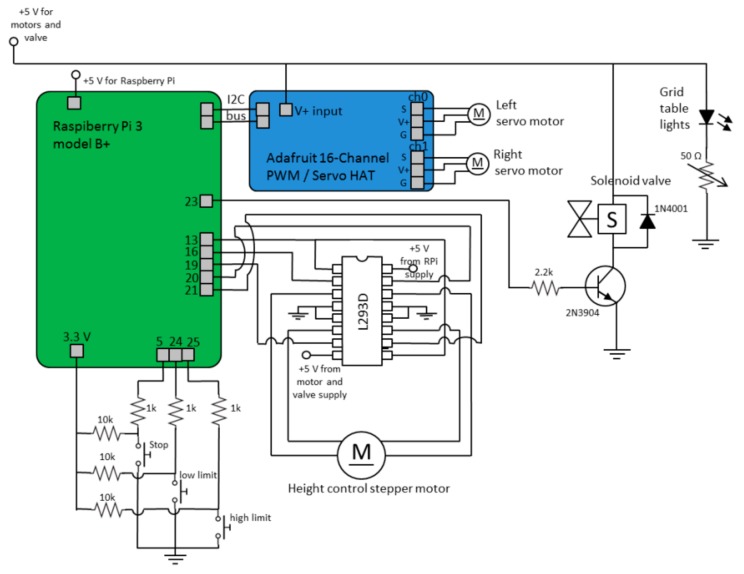
Circuit diagram of the grid-placer robot.

The circuit diagram of the system is shown in Figure 3. The robot is controlled by a Raspberry Pi 3 Model B+ (Premier Farnell Ltd., Leeds, UK) single board computer connected to the motor drivers and control electronics. The *x*-*y* servo motors are controlled via an Adafruit 16 Channel PWM/Servo HAT driver card (Adafruit Industries, New York, NY, USA). The *z*-movement stepper motor is run by a L293D dual H-bridge motor driver. The magnetic valve is controlled by a GPIO (general purpose output/input) output of the Raspberry Pi via a 2N3904 transistor. The Raspberry Pi is powered by its own 5 V power supply, while the motors, valve, and the lights are powered by another 5 V and 2 A-rated USB power supply to prevent interference with the Raspberry Pi.

### 2.5. Computer Vision System

**Figure A1:**
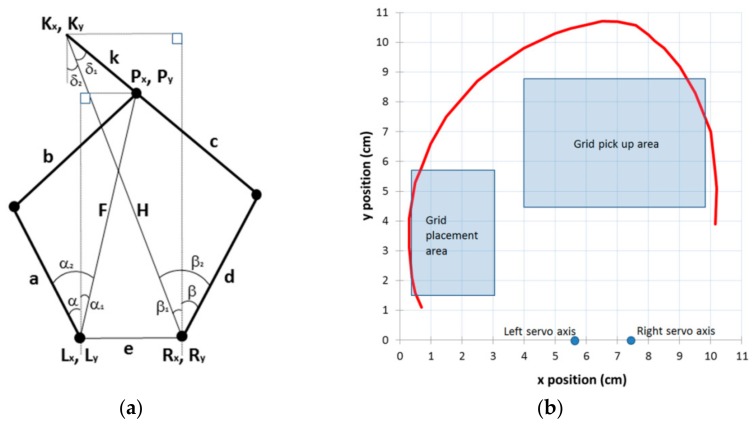
(**a**) Geometry of the five-bar mechanism of the *x*-*y* arm and indication of variables used in calculations. (**b**) The arm range of motion. The arm can reach locations enclosed by the red line. Servo positions and pick-up and placement areas are shown with respect to the range of motion.

The computer vision system that the robot uses to search grids is implemented using a Hama Speak2 web camera. This camera has manual focus that allows focusing to distance necessary for our setup. Other types of web cameras can work too but may need to be modified to adjust the predefined focus. The camera is mounted 85 mm above the grid table on an *x*-*y*-*z* adjustable support fixture built of a 5 mm threaded bar and suitable brackets. The positions of the camera and the *x*-*y* arm assembly are adjusted such that the arm can reach all areas within the field of view of the camera. The range of motion of the robot is calculated in Appendix A and is shown in Figure A1b.

Ambient lights reflecting from the grids can disturb the identification procedure. To prevent this, the grid table has a transparent top with white light-emitting diode (LED) lights underneath. This way, the camera sees the grids against a bright background, making identification and localization reliable. The filter paper holding the grids causes diffusion of the light, resulting in an even illumination. This, however, depends on the number, placement, and brightness of the LEDs used; therefore, we used six LEDs positioned in a 2 × 3 pattern can be used. If more diffusion is needed, a milky plastic piece or sheet may be added.

**Figure 4:**
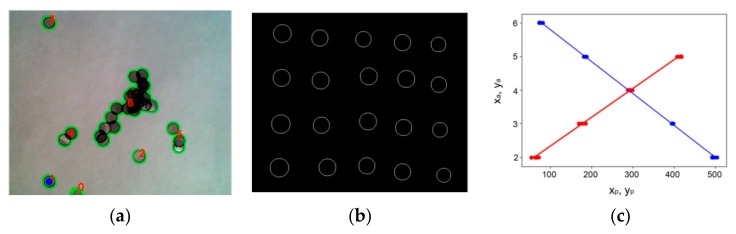
(**a**) A screen capture visualizing the feature selection by the computer vision. Green outlines indicate the found features and blue dot marks a grid selected for pick-up. Features are sorted and numbered according to their area. (**b**) Image showing the result of contour calculation of an image after placing a round marker of 3 mm diameter on a regular grid predetermined coordinates. (**c**) An example plot of calibration data for the transformation between picture coordinates (*x_p_*, *y_p_*) and arm coordinates (*x_a_*, *y_a_*), together with linear fits. The units for the horizontal axis are in pixels, and the units for the vertical axis are in centimeters.

The control software is implemented in Python using the OpenCV Open Source Computer Vision Library version 3.3.0 [4]. To find the grids, a grayscale image of the grid table is taken and smoothed by Gaussian blur. Next, Canny edge detection algorithm is used. Small internal details are filtered out by the OpenCV morphological closing function, and the resulting image is binarized with a threshold set by Otsu’s binarization algorithm. Outer contours of features are located and sorted by contour area. There are two criteria a contour must meet in order to be identified as a single grid: (1) its area must be within given limits, and (2) it must be circular. Circularity is tested by checking that the true area of the contour in pixels is within ±20% of the area of the minimum enclosing circle of the contour calculated by the corresponding OpenCV function. For the first confirmed grid, the centroid coordinates are calculated and passed to the robot arm control routine. If no single grids are found, the remaining contours with area larger than the given grid area are interpreted as heaps of grids, and the centroid coordinates of the first of such contour on the contour list is passed to the robot arm control routine. It is possible that grid heaps have no grids in the calculated centroid position (e.g., circular-shaped heaps), and the program checks whether or not the suggested pick-up coordinates are inside the detected contour before passing the coordinates forward. If the coordinates are outside the heap contour, the surrounding area is investigated by checking the maximum in five steps of 0.8 times the grid diameter in ±*x* and ±*y* directions until coordinates that are on the heap are found. This prevents the robot getting stuck on trying to pick up a heap of grids over and over again from a location where there are no grids. Figure 4a illustrates feature identification by the computer vision program.

The coordinate transformation between the pick-up coordinates found in the image and the corresponding *x*-*y* arm location coordinates is calculated by linear functions $x_{arm}=a_{x}x_{picture}+b_{x}$ and $y_{arm}=a_{y}y_{picture}+b_{y}$. Calculating the constants $a_{x},\;b_{x},a_{y}$ and $b_{y}$ in the abovementioned functions is necessary to calibrate the system. For this, we wrote a program to make the arm place a round marker of 3 mm diameter on a regular grid with predetermined coordinates. At each position, a picture was taken after placing the marker, and the coordinates of the marker centroid were measured. As the final step, these centroid coordinates were compared with the predetermined coordinates that were sent to the arm (Figure 4c).

This is not exactly correct mathematically because the camera deforms pictures taken of objects at close distance, giving them a slight barrel-type curvature. However, the linear assumption was found to be accurate enough to enable reliable grid pick-up. In all tests performed, the arm never missed a grid due to pick-up coordinate tolerance error. For simplicity, the curvature can therefore be neglected. 

### 2.6. Robot Workflow

The user casts the pioloform or formvar film on a microscope slide using standard procedures [1]. The film exactly fits in the free water surface in the trough (in our case 25 mm × 40 mm). The film is then detached in the trough by dipping the slide in the front end of the trough and submerging it slowly while drawing it back as the film gets detached, leaving the glass in the trough. Grids are placed by hand on a filter paper on the grid table, and the robot workflow is started. First, the coordinates for the grid placement on the film are calculated by giving the program the coordinates for the first grid, the grid spacing in *x* and *y*, and the numbers of rows and columns in the intended pattern. Supplementary Materials, Video S1 shows an example of the robot placing a 3 × 3 pattern of grids on the film. The workflow consists of repeating the following steps: (1) determine pick-up target coordinates (Section 2.6); (2) move the *x*-*y* arm to that position; (3) move the arm down to pick up the target and lift up; (4a) if the target is a grid, place it on the next free location on the film and (5) detach by closing the valve on the suction line; (4b) if the target is a heap of grids that needs to be spread apart, move the *x*-*y* arm to the middle of the quadrant of the grid table that has least grids determined by the minimum number of black pixels in the binarized image, lift the arm to the upper limit, and drop the grids picked up from the heap by closing the valve on the suction line and shake quickly to detach grid(s) possibly stuck to the nozzle; (6) move the arm to home position and restart from (1). The workflow is stopped if (1) all predetermined grid positions on the film are filled, (2) it runs out of grids on the table, or (3) the user stops the operation. As a final step, the user lifts the film with the coated grids from the trough as usual using their favorite method, for example, with a piece of parafilm, filter paper, or an address-sticker-coated coverslip.

## 3. Results and Discussion

**Figure 5:**
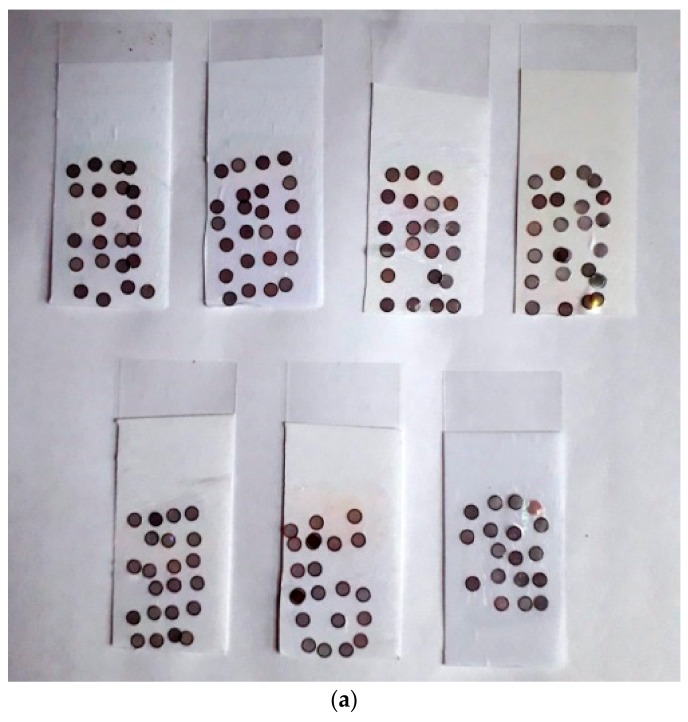

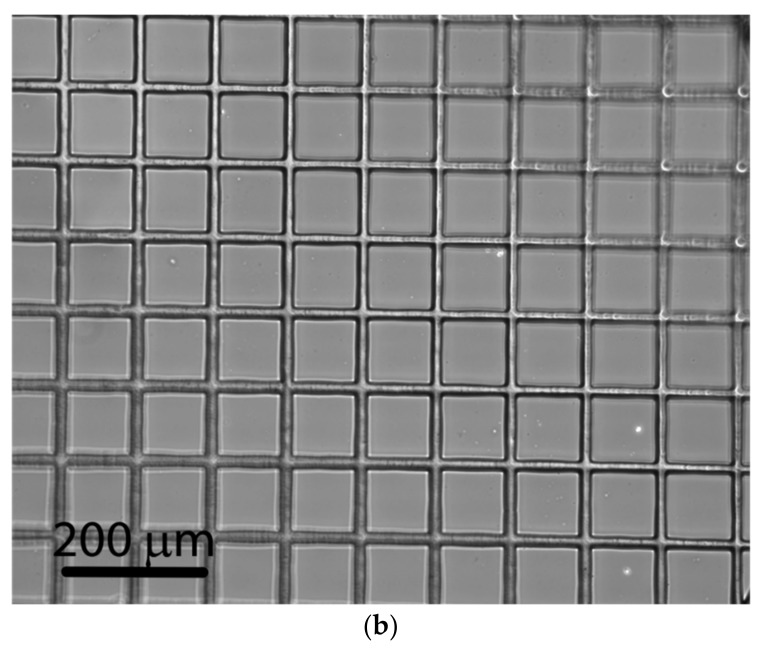
Results of the reliability test. (**a**) Photograph of the films with grids placed in 4 × 6 patterns during the reliability test. In the bottom right film, one row was lost while picking up the film from the trough. (**b**) Typical example of a grid imaged in phase-contrast light microscopy with 10× objective, showing that the film is not damaged. Some small holes are seen in the film, but all grid windows are intact. Occasional small holes in the film can also be seen in grids prepared in the traditional way.

The reliability of the prototype in producing usable grids was tested by letting it place grids on seven films in a 4 × 6 lattice. The grid position spacing in the lattice was 5 mm in both *x* and *y*, which corresponded to an empty space of 2 mm between perfectly placed grids. The films were cast from 1.5% pioloform in chloroform on conventional microscope glass slides and detached in the trough as explained above. Copper grids of 200 and 300 mesh were used. Grids were directly dropped from their container to the grid table with no human arrangement on them beforehand, and the robot was allowed to run without any human intervention or touching of the grids. Grids have sides of shiny and dull appearances, and the robot places the grids with either side randomly facing the film. However, in our experience, the grid side facing the film played no role in the film attachment or in the TEM routine. The resulting films with grid patterns are shown in Figure 5a. The grids were visually inspected under a stereo microscope, phase-contrast light microscope (Zeiss AXIO Vert.A1 with Zeiss LD A-Plan 10×/0.25 Ph1 objective; Carl Zeiss Microscopy GmbH, Jena, Germany). Two grids of each film were imaged in TEM (JEM-1400 Plus, JEOL Ltd., Tokyo, Japan) but no damage was observed, showing that the robot is gentle enough in placing the grids. Representative example of an undamaged film observed in phase-contrast light microscopy is shown in Figure 5b.

Two kinds of mistakes were observed: (1) the grid was not picked up by the nozzle, causing a blank position in the film and (2) an overlapping of grids on the film. The overlap was caused either by grids being so exactly on top of each other on the grid table that they were identified as one or because the nozzle pick-up position was too close to the edge of the grid. In the latter case, the grid was then placed too far off the intended position, overlapping the neighboring grid that might have been misplaced in the opposite direction for the same reason. 

During the reliability test, 138 grids were placed correctly, 26 grids were overlapping, and 13 grids positions were empty. An average of two empty positions per film is acceptable in terms of costs because film itself is very cheap compared to the cost of grids, which is on the range of 0.2–0.3 €/piece. Therefore, an average of two overlapping grids per film is more unfortunate as one or both grids per incidence are lost depending on the degree of overlap. On closer inspection, in eight of the overlapping grids, the two grids were already overlapping during pick-up. In the rest of the cases, only the rims of the grids were overlapping, making at least one of the grids useful. The latter can be prevented by placing the grids slightly further apart, but this will come with a tradeoff of fitting fewer grids on one film. In total, the success rate (determined as the ratio of the number of useful grids to the number of all grids spent) was 90%. Considering that the number of overlap mistakes could be reduced simply by using a sparser pattern, these prototype test results are promising.

In all these tests, it took 6–7 min for the robot to complete the 4 × 6 pattern. The robot is slower than humans, mainly because it takes longer for it to spread a heap of grids to get single grids to pick up. In our experience, a skilled laboratory technician can place the same pattern 2–3 min faster and lose occasional grids by dropping them from the tweezers to the water container. The mistakes can be reduced by a more precise robot arm and adding special sensors or a computer vision system to guide the placement of the grids on the film, but this would have only marginal benefits.

## 4. Conclusions

In this article, we have presented the construction and testing of a prototype of a computer vision-controlled grid-placer robot that is able to automatically place grids during support film preparation. The prototype operates with 90% success rate in preparing useful grids. Even with its current setting, which is slower compared to an experienced laboratory technician, the presented robot concept is helpful in setting a technician free from an ergonomically awkward routine task during TEM grid preparation.

## Figures and Tables

**Table 1:** Parts needed for the construction of the grid-placer robot.

Part	Supplier and Part Number	Approx. Price (€)
Raspberry Pi 3 Model B+ Starter Kit	Premier Farnell (Leeds, UK), RPI3-MODBP-STARTER	55
Adafruit 16-Channel PWM/Servo HAT for Raspberry Pi	Adafruit Industries (New York, NY, USA), Product ID 2327	22
Any web camera; we have Hama Speak2		20
Elite 802 aquarium air pump	e.g., Amazon or eBay	30
Trixie aquarium pump check valve	e.g., Amazon or eBay	2
Dilite aluminum composite sheet about 30 × 30 cm^2^	hardware store	10
6 V valve	eBay	4
0.9 mm syringe needle, plastic syringe, microfluidic connector	laboratory supplies vendors	3
Towerpro SG92R micro servo with two	Amazon	12
old DVD drives		
Transistor 2N3904, diode 1N4001	electronics suppliers	0.1
Dual H-bridge L293D	electronics suppliers	4
10 kΩ, 1 kΩ, 2.2 kΩ, 10 Ω resistors, 50 Ω variable resistor	electronics suppliers	1
USB charger, rating 2 A	electronics suppliers	15
Prototyping board	electronics suppliers	3
Wires, connectors, buttons	electronics suppliers	3
Brackets, bolts, nuts, threaded bar, pneumatic hose	hardware store	10
	Total	approx. €195

PWM: Pulse-width modulation; HAT: hardware attached on top; DVD: digital versatile disc; USB: universal serial bus.

## Supplementary Materials

The following are available online at http://www.mdpi.com/2409-9279/2/1/9/s1, Video S1: The robot arm placing a 3 × 3 grid pattern on pioloform film. S2: Source code of the robot control software.

## Appendix A

The geometry of the arm design is shown in Figure A1a. The left and right servo motors are located at coordinates (*L_x_*, *L_y_*), and (*R_x_*, *R_y_)*, respectively, and the pick-up nozzle is located at (*K_x_*, *K_y_*). Coordinates (*P_x_, P_y_*) indicate the position of the top joint of the arm, and the bar lengths *a*,*b*,*c*,*d*,*e*, and *k* are used as shown in the figure. *F* is the distance from left servo motor to the top joint *P*, and *H* is the distance from right servo motor to the nozzle *K*. Angles α and β are the angles the servo motors are commanded to place the nozzle at the desired position *K*.

The servo angles for moving the nozzle to position (*K_x_*, *K_y_*) are calculated as follows. The left servo angle is $\alpha =\alpha_{2}-\alpha_{1}$, with

(A1) $$\alpha_{1}=\tan^{-1}\;\frac{P_{x}-L_{x}}{P_{y}-L_{y}}$$

with

(A2) $$P_{x}=K_{x}+k\sin\left(\delta_{1}+\delta_{2}\right)=K_{x}+k\sin\left(\delta_{1}+\beta_{1}\right)\;\;P_{y}=K_{y}-k\cos\left(\delta_{1}+\delta_{2}\right)=K_{y}+k\cos\left(\delta_{1}+\beta_{1}\right)$$

The angles *δ*_1_ and β_1_ are calculated as follows:

(A3) $$\delta_{1}=\cos^{-1}\;\frac{{\left(c+k\right)}^{2}+H^{2}-d^{2}}{2H\left(c+k\right)}\;\;\mathrm{with}\;\;\;H=\sqrt{{\left(R_{x}-K_{x}\right)}^{2}+{\left(R_{y}-K_{y}\right)}^{2}}$$

and

(A4) $$\beta_{1}=\tan^{-1}\;\frac{R_{x}-K_{x}}{K_{y}-R_{y}}$$

Angle $\alpha_{2}$ is calculated from

(A5) $$\alpha_{2}=\cos^{-1}\;\frac{a^{2}+F^{2}-b^{2}}{2Fa}\;\;\mathrm{with}\;\;F=\sqrt{{\left(P_{x}-L_{x}\right)}^{2}+{\left(P_{y}-L_{y}\right)}^{2}}$$

using coordinates of *P* from Equation (A2).

Right servo angle is $\beta =\beta_{2}\;-\beta_{1}$, where $\beta_{1}$ comes from Equation (A4), and $\beta_{2}$ is calculated from

(A6) $$\beta_{2}=\cos^{-1}\;\frac{d^{2}+H^{2}-{\left(c+k\right)}^{2}}{2Hd}$$

with *H* from Equation (A3).

The range of motion of the arm is visualized in Figure A1b, which shows results calculated by numerically solving the above equations for *K_x_*, and *K_y_* at extreme motor positions. The grid pick-up area and the placement area on the film are positioned to maximize their areas while still keeping them well inside the range of motion. The camera is adjusted to see only the pick-up area.
